# Exposure to Air Pollutants Increases the Risk of Chronic Rhinosinusitis in Taiwan Residents

**DOI:** 10.3390/toxics10040173

**Published:** 2022-04-01

**Authors:** Shih-Wei Chen, Han-Jie Lin, Stella Chin-Shaw Tsai, Cheng-Li Lin, Chung Y. Hsu, Tsai-Ling Hsieh, Chuan-Mu Chen, Kuang-Hsi Chang

**Affiliations:** 1 Department of Life Sciences and Ph.D. Program in Translational Medicine, National Chung Hsing University, Taichung 402, Taiwan; eddielodz@gmail.com; 2 Department of Otolaryngology, Tungs’ Taichung MetroHarbor Hospital, Taichung 435, Taiwan; commandity@gmail.com (H.-J.L.); tsaistella111@gmail.com (S.C.-S.T.); a92015t@gmail.com (T.-L.H.); 3 Rong Hsing Research Center for Translational Medicine, College of Life Sciences, National Chung Hsing University, Taichung 402, Taiwan; 4 Management Office for Health Data, China Medical University Hospital, Taichung 404, Taiwan; orangechengli@gmail.com; 5 Graduate Institute of Clinical Medical Science, China Medical University, Taichung 404, Taiwan; hsucy63141@gmail.com; 6 Department of Medical Research, Tungs’ Taichung MetroHarbor Hospital, Taichung 435, Taiwan; 7 The iEGG and Animal Biotechnology Center, National Chung Hsing University, Taichung 402, Taiwan; 8 Center for General Education, China Medical University, Taichung 404, Taiwan; 9 General Education Center, Jen-Teh Junior College of Medicine, Nursing and Management, Miaoli 356, Taiwan

**Keywords:** air pollution, chronic rhinosinusitis, longitudinal health insurance database, Taiwan air quality-monitoring database

## Abstract

Air pollution triggers a tissue-specific inflammatory response. However, studies on the association between exposure to air pollutants and chronic rhinosinusitis (CRS) risk remain limited. Thus, we conducted this nationwide study to define the association between air pollution and CRS. We used the Longitudinal Health Insurance Database (LHID) and Taiwan Air Quality-Monitoring Database (TAQMD) to conduct a population-based cohort study. Study participants were recruited from the LHID, a data subset of the National Health Insurance Research Database that randomly sampled one million individuals. TAQMD has been an air pollutant database since 1998. In univariate and multivariate Cox proportional hazards regression models, adjusted hazard ratios (aHRs) and 95% CIs of CRS in five air pollutants were accounted. We adjusted for age, sex, urbanization level, insurance fee, comorbidities, and pollutant levels in the multivariate model. The total number of participants enrolled in this study was 160,504. The average age was 40.46 ± 14.62 years; males constituted 43.8% of the total participants. The percentages of alcoholism, tobacco dependence, and COPD were 1.5%, 2.8%, and 28.3%, respectively. After adjustment for age, sex, urbanization level, insurance fee, and comorbidities, the highest levels of air pollutants, including PM_2.5_ (aHR = 1.14, 95% CI = 1.06–1.22), NO_2_ (aHR = 1.07, 95% CI = 1.00–1.15), and PM_10_ (aHR = 1.13, 95% CI = 1.05–1.21) had a significantly greater CRS risk; we selected the lower concentration as the reference but did not correlate with CO. We found a significantly increased risk of CRS in residents with air pollutant exposure.

## 1. Introduction

In Taiwan, industrial emission is the main source of air pollution [1]. The following sources are traffic emissions, coal and other fossil fuel combustion, and waste incinerators [2,3]. The health effects of long-term exposure to air pollution have received increasing attention in recent decades. A previous study indicated that there were approximately 3.3 million premature deaths per year worldwide caused by outdoor exposure to air pollution, mostly in Asia [4]. Extensive evidence has shown strong associations between air pollution exposure and several diseases, such as cardiovascular, cerebrovascular, and autoimmune diseases, as well as bone degeneration, hearing loss, malignant tumors, and issues pertaining to child development [5,6,7,8,9,10,11,12,13]. Chronic exposure to air pollution may trigger tissue-specific inflammation [14,15,16].

Chronic rhinosinusitis (CRS) is a disease with chronic sinonasal inflammation [17]. A national population-based study in Korea showed a positive correlation between PM_10_ concentrations and CRS risk; however, no association was observed between the other four pollutants [18]. Additionally, a time-series study in China found that after adjusting the exposure–response relationship curve between six investigated air pollutants and chronic sinusitis in hospital outpatients, a sharp rising curve at low concentrations followed by either a gentle turn in PM_2.5_, PM_10_, SO_2_, or flat to declining trends in NO_2_ and CO at higher concentrations, respectively, were observed. There was no obvious linear relationship between O_3_ and chronic sinusitis in outpatients [19]. A study assessing the correlation between exposure to fine particulate air pollution and self-reported sinusitis using two national datasets disclosed an increased overall aHR, which was not found within the Hispanic group stratified by subgroup analysis [20]. Other studies have shown that air pollution could be positively linked to the incidence and prevalence of CRS, which could have stimulated increased symptomatology and the need for endoscopic sinus surgery [21,22,23]. A questionnaire-based cross-sectional study reported that occupational exposure to gases, fumes, dust, or smoke increased the overall risk for CRS [24]. In addition, Min et al. conducted a nationwide survey on the prevalence and risk factors of chronic sinusitis in Korea. They disclosed that economic activity, air pollution, crowded environments, and allergic rhinitis could be attributed to chronic sinusitis, while other environmental factors such as birthplace, education, and different social classes according to occupation had no influence on its prevalence [25]. Moreover, research on particulate matter has been conducted on many occasions. One recent study found that an increase in PM_2.5_ correlated with a 1.89-fold increase in the requirement for surgery in CRS without nasal polyps (CRSsNPs) [26]. In contrast, no association was identified in two European longitudinal cohort studies between an increased exposure to PM_2.5_ and rhinitis [27]. In summary, we consider that the current clinical data demonstrates a slightly positive correlation between specific air pollutants and CRS; however, the definite connection of both remains to be explored.

Thus, we merged two national databases with more than 10 years follow-up to assess the relationship between exposure to gaseous pollutants and particulate matter and the risk of CRS.

## 2. Materials and Methods

### 2.1. Data Source

We used the Longitudinal Health Insurance Database (LHID) and the Taiwan Air Quality-Monitoring Database (TAQMD) to conduct a population-based cohort study. Study participants were recruited from the LHID 2000, a data subset of the National Health Insurance Research Database (NHIRD), which randomly sampled one million individuals; NHIRD covers almost all of Taiwan’s population (99%) since 1995 and includes information on medical records. These records include the history of outpatients, inpatient visits, medical prescriptions, and other related services. The diagnostic code criteria were derived from the coding system of the International Classification of Diseases, Ninth Revision, Tenth Revision, Clinical Modification (ICD-9-CM, ICD-10-CM). To ensure privacy, the identification numbers were made anonymous upon documentation; informed consent was also obtained. Moreover, TAQMD has been recording data about air pollution since 1998; it was also in charge of the Taiwan Environmental Protection Administration (EPA). Air quality data were collected from 74 air quality monitoring stations in Taiwan. We used the residential areas of the LHID insurant to link the nearest air quality monitoring station information with individuals found in the TAQMD. The residential address was not available from LHID. The definition of residential areas was based on the location of the medical institution with treatment records of the common cold (ICD-9-CM code 460). Based on the residential areas and the site of Air Quality Monitoring stations, the LHID and the TAQMD were connected [6,7,8,28]. This study was approved by the Central Regional Research Ethics Committee of China Medical University, Taichung, Taiwan (CMUH104-REC2-115(CR-6)).

### 2.2. Study Participants

We enrolled participants with no history of CRS (ICD-9-CM code 473.9) and who lived in places where an air quality-monitoring station was used during the study period (January 1998 to 31 December 2011). The index date was defined as 1 January 2000 and the follow-up was until the development of CRS or the end of the database. We excluded participants with missing information, such as age, sex, urbanization, and air pollution data, and those who were diagnosed with CRS before the index date. In accordance with inclusion and exclusion criteria, we enrolled 160,504 residents.

### 2.3. Exposure Collection and Outcome Measurement

The main air pollutants were as follows: SO_2_, CO, NO_2_, PM_2.5_, and PM_10_; these were classified into three levels from lowest to highest. All air pollutant data were provided by a monitoring station in Taiwan; the daily average air pollutant concentration for each participant was calculated from 2000 to 2011. The pollutant concentrations were available from the Environmental Protection Administration Executive Yuan, R.O.C. (Taiwan) [29]. The primary outcome of this study was the development of CRS.

### 2.4. Confounding Factors

Age, insurance fee, urbanization, and comorbidities were included as demographic factors in this study. Furthermore, comorbidities such as alcoholism (ICD-9-CM: 303), tobacco dependence (ICD-9-CM: 305.1), and chronic obstructive pulmonary disease (ICD-9-CM:490–496) were presented as confounding factors.

### 2.5. Statistical Analysis

Continuous and categorical data were presented as mean ± SD and number (%); different levels of air pollutants were expressed as means and standard deviations. We calculated the incidence density rate of the CRS (per 10,000 person-years) according to the level of air pollutants. Poisson regression models were used to estimate the incidence rate ratios (IRR) and 95% confidence intervals (CIs) of CRS events associated with moderate and high-level groups, as compared with the lowest level group. In the univariate and multivariate Cox proportional hazards regression models, adjusted hazard ratios (aHRs) and 95% CIs of CRS in the five air pollutants were accounted. We adjusted for age, sex, urbanization level, insurance fee, comorbidities, and pollutant levels in the multivariate model. Statistical analyses were performed using SAS version 9.4 (SAS Institute Inc., Carey, NC, USA).

## 3. Results

Table 1 presents the demographic characteristics of the participants and shows the distribution of the different levels of air pollutants. The total number of participants enrolled in this study was 160,504. The average age was 40.46 ± 14.62 years; males constituted 43.8% of the total participants. The percentages of alcoholism, tobacco dependence, and COPD were 1.5%, 2.8%, and 28.3%, respectively. Among all levels of insurance fee, the rate of participants in the second level was the highest (32.4%); participants who lived in the highest urbanization accounted for the highest proportion of the population in this study (34.5%). Approximately 30% of the participants were distributed in each level of air pollutant, including SO_2_, CO, NO_2_, PM_2.5_, and PM_10_.

Table 2 shows the risks for CRS in participants exposed to the three levels of concentration of the five air pollutants. When comparing the lowest SO_2_ concentration, participants exposed to the moderate and high concentrations had IRRs of 1.07 and 0.79, respectively. When comparing the CO concentration, participants exposed to the moderate and high concentrations had IRRs of 1.09 and 0.99, respectively. When comparing the lower concentration of PM_2.5_, participants exposed to the moderate and high concentrations had IRRs of 1.18 and 1.16, respectively. When comparing the NO_2_ concentration, participants exposed to the moderate and high concentrations had IRRs of 1.06 and 1.02, respectively. When comparing the lower concentration of PM_10_, participants exposed to the moderate and high concentrations had IRRs of 1.17 and 1.16, respectively.

In the multivariate Cox regression analysis, we estimated the aHR of CRS (Table 3). The results showed that a 1-year exposure to any air pollutant concentration, including SO_2_, CO, NO_2_, PM_2.5_, and PM_10_, significantly increased the risk of CRS after controlling for risk factors. Compared to the non-alcoholic group, the alcoholic group had a decreased risk of CRS after controlling for age, sex, urbanization level, insurance fee, and comorbidities, and were adjusted separately for each air pollutant concentration, such as SO_2_ (aHR = 0.65, 95% CI = 0.50–0.85), CO (aHR = 0.65, 95% CI = 0.50–0.84), PM_2.5_ (aHR = 0.65, 95% CI = 0.50–0.85), NO_2_ (aHR = 0.65, 95% CI = 0.50–0.84), and PM_10_ (aHR = 0.65, 95% CI = 0.50–0.85). For the non-tobacco and tobacco groups, after controlling for risk factors such as age, sex, urbanization level, insurance fee, and comorbidities, the results were adjusted separately for each air pollutant concentration, including SO_2_, CO, NO_2_, PM_2.5_, and PM_10_; no significant correlation between with and without tobacco dependence for the risk of CRS was observed. When selecting the non-COPD group as a reference, participants in the COPD group had a greater CRS risk after controlling for age, sex, urbanization level, insurance fee, and comorbidities; the results were adjusted separately for each air pollutant concentration, including SO_2_ (aHR = 2.23, 95% CI = 2.10–2.36), CO (aHR = 2.24, 95% CI = 2.12–2.37), PM_2.5_ (aHR = 2.23, 95% CI = 2.11–2.37), NO_2_ (aHR = 2.24, 95% CI = 2.11–2.37), and PM_10_ (aHR = 2.23, 95% CI = 2.11–2.37). Furthermore, participants in the moderate and high levels of insurance fee showed a significantly higher CRS risk than those in the lower levels after controlling for age, sex, urbanization level, insurance fee, and comorbidities; the results were adjusted separately for each air pollutant (SO_2_, CO, NO_2_, PM_2.5_, and PM_10_). Male participants had a higher CRS risk than women after controlling for age, sex, urbanization level, insurance fee, and comorbidities; the results were adjusted separately for each air pollutant concentration, including SO_2_ (aHR = 1.22, 95% CI = 1.15–1.29), CO (aHR = 1.22, 95% CI = 1.15–1.29), PM_2.5_ (aHR = 1.22, 95% CI = 1.15–1.29), NO_2_ (aHR = 1.22, 95% CI = 1.15–1.29), and PM_10_ (aHR = 1.22, 95% CI = 1.15–1.28). Furthermore, SO_2_ (aHR = 1.08, 95% CI = 1.01–1.15), CO (aHR = 1.13, 95% CI = 1.06–1.21), PM_2.5_ (aHR = 1.18, 95% CI = 1.10–1.26), NO_2_ (aHR = 1.09, 95% CI = 1.02–1.17), and PM_10_ (aHR = 1.17, 95% CI = 1.10–1.26) concentrations were significantly higher after controlling for age, sex, urbanization level, insurance fee, and comorbidities. After age, sex, urbanization level, insurance fee, and comorbidities adjustment, the highest levels of air pollutants included PM_2.5_ (aHR = 1.14, 95% CI = 1.06–1.22), NO_2_ (aHR = 1.07, 95% CI = 1.00–1.15), and PM_10_ (aHR = 1.13, 95% CI = 1.05–1.21); these had significantly greater CRS risks as compared to the lower reference concentrations.

## 4. Discussion

The results demonstrated that in the 11-year period, moderate levels of NO_2_, PM_2.5_, and PM_10_ were attributable to elevated CRS risks, with only minimal HR elevations of 1.09 (95% CI, 1.02–1.17), 1.18 (95% CI, 1.10–1.27), and 1.17 (95% CI, 1.10–1.25), respectively (Table 2). No statistical significance was observed for either the SO_2_ or NO_2_ concentrations. Individuals with higher HRs resided in the second highest urbanization area and were exposed to moderate levels of air pollutants (Table 3). However, exposure to high levels of air pollutants in the highest urbanization area did not pose a higher risk for developing CRS. Conversely, COPD had detrimental effects on the exacerbation of CRS, which is consistent with previous results [30,31]. In this regard, our results showed only a weak association between air pollutants and CRS at moderate concentration levels. Thus, further research is needed to elucidate this relationship.

Traffic related air pollutants (TRAP), such as diesel exhaust particles (DEPs), NO_2_, particulate matter, and cigarette smoke are common environmental factors that may correlate with CRS. However, the fundamental pathologic mechanisms of CRS remain unclear [32,33]. Several studies have connected air pollution exposure to the pathologic mechanisms of CRS, including systemic inflammation with elevated oxidative stress, disruption of the epithelial barrier, and ciliary dysfunction. Epithelial cells release pro-inflammatory cytokines upon exposure to air pollutants. These circulating inflammatory markers further trigger the local recruitment of inflammatory cells and increase oxidative stress. Previous studies have examined biomarkers that sustain systemic inflammation after PM exposure, including tumor necrosis factor (TNF)-α, interleukin (IL)-6, and IL-8 [34,35]. In an in vivo study wherein, mice were administered DEP-impregnated ragweed pollen, the results revealed disrupted tight junction integrity, thereby leading to sinonasal epithelial barrier compromise and increased levels of IL-6 and IL-8 [36,37]. The effect of DEPs on the enhanced expression of IL-6 and IL-8 in nasal fibroblasts was further confirmed by ex vivo examination of the inferior turbinates [38]. Furthermore, experimental in vivo ambient PM_2.5_ exposure has been performed in mice. Inhalation of PM_2.5_ induced sinonasal inflammatory cells, including macrophages, neutrophils, and eosinophils, accompanied by epithelial barrier dysfunction and the increased expression of pro-inflammatory cytokines, such as IL-1β, IL-13, and eotaxin-1 [39]. Furthermore, increased levels of reactive oxygen species (ROS), decreased production of counter-regulatory superoxide dismutase, catalase, and glutathione peroxidase, and epithelial cellular injury were also observed [40,41]. These clinical observations may help explain the pathogenesis of CRS; however, the explicit mechanism regarding its inflammatory pathophysiology could not be fully delineated for heterogeneity.

The current understanding of CRS has changed the disease classification from a phenotype (i.e., CRS with nasal polyps (CRSwNPs), without nasal polyps, and eosinophilic CRS) into endotypes (either type 2 or non-type 2 inflammation response) [42]. A former analysis of cytokine profiles, transcription factors, and cellular responses in nasal polyps revealed that white races with CRSwNPs presented elevated levels of IL-5 (T_h_2 cytokine) and GATA-3 (T_H_2 transcription factor) along with eosinophilic inflammation; Chinese participants with CRSwNPs demonstrated a T_H_1/T_H_17 pattern with neutrophilic inflammation, as compared to the control group [43]. Another study found that the central south Chinese population with CRSsNPs had higher levels of IFN-γ, a regulatory cytokine expressed in T_H_1 inflammation, whereas only a small group with eosinophilic CRSwNPs exhibited enhanced GATA-3 and IL-5 expression [44]. Wang et al. described a significant diversity in T_H_ cytokine and inflammatory signatures between regions of three continents: Asia (China and Japan), Europe (Benelux and Germany), and Australia. Tissues from CRSwNPs in Europe and Australia were characterized by a stronger expression of type 2 inflammation (associated with T_H_2 mediated pathway) than in Asian patients. However, in Asia, the expression patterns varied from a low type 2 expression in Chengdu/China to a moderate expression in Beijing and Japan [45]. These findings were further supported by a longitudinal cohort study by Chen et al., wherein an association between selective ambient PM_2.5_ and an increased percentage of neutrophils and IL-8 in the nasal cavity of school-aged children was observed [46]. Accordingly, the pathogenesis of CRS varies across races and investigated areas; it may also be influenced by various environmental factors. The association between air pollution and CRS in this study remains unclear. As a result, our current research may indicate evidence that the inflammatory patterns in most Asian CRS with TH1/TH17 are not comparable with previous understandings of systemic cytokine responses with higher levels of TNF-α, IL-1, IL-6, and IL-8. We consider that there was only a weak association between the air pollutant exposure and the pathogenesis of CRS and a further randomized study may be needed.

There are some limitations in this study. First, the exposure assessment utilized a combination approach of two databases: the NHIRD and TAQMD by the Taiwan EPA. Each air quality monitoring station covered a nearby residential area of less than 250 persons for every 150 square kilometers of land surface area [47]. The air quality data were collected from the nearest station of the registered hospitals where the participants were diagnosed with CRS. Thus, the measured levels of air pollutants could be different from the real situation; commuting patients would be exposed to varying air quality across different places, which may be inconsistent with those visiting the hospitals. Second, Because of its overlapping clinical presentations with other diseases such as COPD, the accuracy of diagnosing CRS may be limited, and a definitive causality between air pollution exposure and the following diagnosis of CRS could not be stratified due to a lack of temporal linkage by the current method. Third, a subgroup analysis of CRS according to endotypes could not be applied to the study; other confounding factors were not taken into account for risk adjustments. Finally, the resident may have not been enrolled if they had no treatment records of the common cold during study period and this may have caused an underestimation of the association between air pollutant exposure and CRS.

## 5. Conclusions

From our perspective, the present study provides an insight into the impact of the long-term exposure to air pollutants on CRS. Despite the research limitations, well-structured clinical research with complete information of healthy behaviors and the level of personal exposure to air pollution is warranted to further elucidate this causal relationship.

## Figures and Tables

**Table 1:** The demographic data of the study participants.

*n* = 160,504	Subgroups	*n*	%
Age (mean, SD)		40.46	14.62
Follow up years (mean, SD)		11.71	0.93
Male		70,352	43.8
Alcoholism		2477	1.5
Tobacco dependence		4503	2.8
COPD		45,407	28.3
Levels of insurance fee	Lowest	25,165	15.7
	2nd	51,927	32.4
	3rd	37,417	23.3
	Highest	45,995	28.7
Urbanization	Highest	55,306	34.5
	2nd	52,152	32.5
	3rd	27,215	17.0
	Lowest	25,831	16.1
SO_2_	Low	52,037	32.4
	Moderate	62,730	39.1
	High	45,737	28.5
CO	Low	56,720	35.3
	Moderate	51,023	31.8
	High	52,761	32.9
PM_2.5_	Low	53,235	33.2
	Moderate	53,586	33.4
	High	53,683	33.4
NO_2_	Low	55,071	34.3
	Moderate	54,227	33.8
	High	51,206	31.9
PM_10_	Low	59,636	37.2
	Moderate	50,209	31.3
	High	50,659	31.6

**Table 2:** Incidence and incidence rate ratio of CRS for the three levels of pollutant exposure.

Pollutants	Levels	*n* of CRS	Person Years	Incidence Rate	Incidence Rate Ratio	95% CI	
SO_2_	Low	1770	607,762	2.91	1.00		
	Moderate	2271	737,033	3.08	1.07	1.00	1.13
	High	1216	534,141	2.28	0.79	0.73	0.85
CO	Low	1801	665,244	2.71	1.00		
	Moderate	1794	599,002	2.99	1.09	1.03	1.17
	High	1662	614,690	2.70	0.99	0.93	1.06
PM_2.5_	Low	1529	624,050	2.45	1.00		
	Moderate	1864	628,477	2.97	1.18	1.10	1.26
	High	1864	626,409	2.98	1.16	1.09	1.24
NO_2_	Low	1756	645,644	2.72	1.00		
	Moderate	1854	636,774	2.91	1.06	0.99	1.13
	High	1647	596,519	2.76	1.02	0.95	1.09
PM_10_	Low	1733	699,227	2.48	1.00		
	Moderate	1766	589,134	3.00	1.17	1.10	1.25
	High	1758	590,575	2.98	1.16	1.08	1.24

Incidence rate (1000 person-years); CI, confidence interval; The annual average air pollutant concentrations were categorized into 3 groups based on tertiles for each air pollutant.

**Table 3:** Adjusted HR of CRS in the moderate and high concentration groups compared to that in the low concentration group.

Covariates	SO_2_			CO			PM_2.5_			NO_2_			PM_10_		
	aHR	95% CI		aHR	95% CI		aHR	95% CI		aHR	95% CI		aHR	95% CI	
Age	0.998	0.996	1.00	0.998	0.996	1.00	0.998	0.996	1.00	0.998	0.996	1.00	0.998	0.996	1.00
Sex															
Female	1.00			1.00			1.00			1.00			1.00		
Male	1.22	1.15	1.29	1.22	1.15	1.29	1.22	1.15	1.29	1.22	1.15	1.29	1.22	1.15	1.28
Alcoholism															
No	1.00			1.00			1.00			1.00			1.00		
Yes	0.65	0.50	0.85	0.65	0.50	0.84	0.65	0.50	0.85	0.65	0.50	0.84	0.65	0.50	0.85
Tobacco dependence															
No	1.00			1.00			1.00			1.00			1.00		
Yes	1.06	0.92	1.23	1.07	0.92	1.24	1.06	0.92	1.23	1.07	0.92	1.24	1.06	0.92	1.24
COPD															
No	1.00			1.00			1.00			1.00			1.00		
Yes	2.23	2.10	2.36	2.24	2.12	2.37	2.23	2.11	2.37	2.24	2.11	2.37	2.23	2.11	2.37
Levels of insurance fee															
Lowest	1.00			1.00			1.00			1.00			1.00		
2nd	1.11	1.02	1.21	1.11	1.02	1.21	1.11	1.02	1.22	1.11	1.02	1.22	1.11	1.02	1.22
3rd	1.08	0.99	1.19	1.10	1.00	1.20	1.09	0.99	1.20	1.10	1.00	1.20	1.09	1.00	1.20
Highest	1.21	1.10	1.32	1.21	1.11	1.32	1.21	1.11	1.33	1.21	1.11	1.32	1.21	1.11	1.33
Urbanization															
Highest	1.00			1.00			1.00			1.00			1.00		
2nd	1.16	1.09	1.24	1.11	1.04	1.19	1.10	1.03	1.17	1.12	1.05	1.20	1.09	1.02	1.17
3rd	1.08	0.99	1.17	1.00	0.92	1.09	0.99	0.91	1.08	1.01	0.93	1.10	0.99	0.91	1.07
Lowest	1.05	0.97	1.14	1.06	0.97	1.15	1.03	0.94	1.12	1.08	0.99	1.18	1.02	0.94	1.11
Pollutant levels															
Low	1.00			1.00			1.00			1.00			1.00		
Moderate	1.08	1.01	1.15	1.13	1.06	1.21	1.18	1.10	1.26	1.09	1.02	1.17	1.17	1.10	1.26
High	0.79	0.73	0.85	1.03	0.96	1.11	1.14	1.06	1.22	1.07	1.00	1.15	1.13	1.05	1.21

cHR, crude hazard ratio; aHR, adjusted hazard ratio, adjusted for age, sex, alcoholism, tobacco dependence, COPD, levels of insurance fee, urbanization, and pollutant levels; CI, confidence interval; The annual average air pollutant concentrations were categorized into 3 groups based on tertiles for each air pollutant.

## Data Availability

Data are available from the NHIRD published by Taiwan National Health Insurance Bureau. Due to the ‘Personal Information Protection Act’, data cannot be made publicly available (http://nhird.nhri.org.tw/en/index.html) (Accessed on 1 December 2021).
